# Differential contribution of T‐type voltage‐gated calcium channels to vascular reactivity in the aorta and renal artery of healthy rabbits

**DOI:** 10.1113/EP093044

**Published:** 2025-10-27

**Authors:** Andrea Suarez, Sol Guerra‐Ojeda, Manuel Zarzoso, Marta Serna‐García, Jose M. Vila, Eva Serna, Maria D. Mauricio

**Affiliations:** ^1^ Department of Physiology, InVas and ModulAhR Groups, Facultat de Medicina i Odontologia Universitat de Valencia, INCLIVA Biomedical Research Institute Valencia Spain; ^2^ Department of Physiotherapy Faculty of Physiotherapy University of Valencia Valencia Spain; ^3^ Department of Dentistry Faculty of Health Sciences Universidad Europea de Valencia Valencia Spain; ^4^ Centro de Investigación Biomédica en Red Fragilidad y Envejecimiento Saludable CIBERFES Valencia Spain; ^5^ Centro de Investigación Biomédica en Red Enfermedades Cardiovasculares CIBERCV Valencia Spain

**Keywords:** aorta, endothelium, eNOS, renal artery, T‐type voltage‐gated calcium channels, vascular reactivity

## Abstract

T‐type voltage‐gated calcium channels (VGCCs) are comparatively understudied in large conduit artery function relative to the well‐characterized L‐type channel subtype. This study investigates the vascular roles of T‐type VGCCs in the aorta and renal artery of healthy rabbits using functional reactivity assays and immunofluorescence and further elucidates the interaction between T‐type VGCC activity and the nitric oxide (NO) signalling pathway. T‐type VGCCs contributed to contractile responses induced by phenylephrine and angiotensin II in both arteries. Moreover, in the renal artery, the contribution of T‐type VGCCs in response to phenylephrine increased in the absence of NO and was associated with endothelium‐dependent vasodilation. Immunofluorescence analysis revealed co‐localization of Ca_V_3.1 with endothelial nitric oxide synthase in the renal artery, but not in the aorta, suggesting a vasodilatory role in renal circulation. These findings highlight vascular bed‐specific functions of T‐type VGCCs and their interaction with endothelial pathways in the renal artery.

## Abstract (highlights)

- **What is the central question of this study?**
  Do T‐type voltage‐gated calcium channels (VGCCs) regulate vascular tone in large conduit arteries, and is their function artery‐specific or influenced by endothelial factors?
- **What is the main finding and its importance?**
  T‐type VGCCs contribute to vasoconstriction in both the aorta and renal artery and mediate vasodilation exclusively in the renal artery. Their activity is enhanced under nitric oxide (NO) deficiency, and Cav3.1 colocalizes with eNOS only in the renal artery. This highlights a functional, artery‐specific role for T‐type VGCCs in conduit vessels, with implications for vascular regulation and endothelial signaling.

## Graphical abstract

**Abstract figure** T‐type VGCCs contribute to the contraction of the rabbit aorta and modulate both contraction and vasodilation in the rabbit renal artery. α_1_, alpha 1 adrenergic receptor; ATR, angiotensin II receptor; Ang II, angiotensin II; CaM, calmodulin; Cav_3_, T‐type voltage‐gated Ca^2^⁺ channels; cGMP, cyclic guanidine monophosphate; EC, endothelial cell; eNOS, endothelial nitric oxide synthase; GTP, guanidine triphosphate; MLC, myosin light chain; MLCK, myosin light chain kinase; NO, nitric oxide; Phe, phenylephrine; sGC, soluble guanylate cyclase; VSMC, vascular smooth muscle cell.

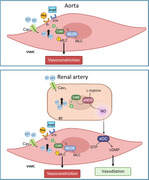

## 1 INTRODUCTION

The main pathway for calcium entry in vascular smooth muscle cells (VSMCs) is mediated by L‐type voltage‐gated calcium channels (VGCCs), which play a pivotal role in the maintenance of vascular tone (Moosmang et al., 2003). However, increasing evidence indicates that T‐type VGCCs, particularly in small resistance arteries, also contribute to the fine‐tuning of vascular function (Abd El‐Rahman et al., 2013; Hansen et al., 2001; Navarro‐Gonzalez et al., 2009).

T‐type VGCCs activate at lower depolarization levels than L‐type VGCCs, have faster kinetics, and are primarily found in the nervous system and cardiac pacemaker cells, playing a crucial role in cellular excitability (Todorovic & Lingle, 1998; Weiss & Zamponi, 2019). Currently, three subtypes of T‐type VGCCs are known: Ca_V_3.1, Ca_V_3.2 and Ca_V_3.3. In the cardiovascular system, the predominant subtypes are Ca_V_3.1 and Ca_V_3.2 (Braunstein et al., 2008; Kuo et al., 2010; Tomida et al., 2024; Wu et al., 2003). Their activation has been associated with vasoconstriction in rat and human cerebral arteries (Abd El‐Rahman et al., 2013; Navarro‐Gonzalez et al., 2009; Thuesen et al., 2017), rat renal microvessels (Hansen et al., 2001), rat mesenteric and human subcutaneous arteries (Ball et al., 2009), and other resistance vessels (Björling et al., 2013; Braunstein et al., 2008; Jensen & Holstein‐Rathlou, 2009; Kuo et al., 2010; Poulsen et al., 2011), or vasodilation in mice pulmonary artery (Gilbert et al., 2017), mesenteric artery (Fan et al., 2018) and other resistance vessels (Chen et al., 2003; El‐Lakany et al., 2023; Harraz et al., 2014; Poulsen et al., 2011; Svenningsen et al., 2014; Thuesen et al., 2014). Concerning conduit arteries, T‐type VGCCs appear to have a limited role in aorta (Ball et al., 2009) or have been associated with vasoconstriction in human mammary artery (Thuesen et al., 2017). To date, there is no clear evidence supporting a direct vasodilatory role for T‐type VGCCs in conduit arteries.

Collectively, these studies highlight the expression of T‐type VGCCs across resistance vascular beds and their heterogeneous contributions to vascular tone and reactivity. Nevertheless, they also reveal a notable gap in our understanding of T‐type VGCC function in large conduit arteries. In this context, the present study investigates the aorta and renal arteries of healthy rabbits to provide novel insights into the functional role of these channels and to broaden our understanding of their contribution to vascular regulation beyond the microvascular domain. Moreover, it has been suggested that nitric oxide (NO) deficiency may activate T‐type VGCCs (Howitt et al., 2013, 2015; Ng et al., 2024; Smith et al., 2020). Therefore, since endothelial dysfunction in conduit arteries is mainly associated with a deficit of NO, these channels might become more active in the context of cardiovascular diseases. We hypothesize that, under physiological conditions, NO downregulates the function of T‐type VGCCs in conduit arteries. We aim to determine the role of T‐type VGCCs in the contractile response to phenylephrine in the absence and presence of NO and to angiotensin II in the aorta and renal artery of healthy rabbits. Additionally, we aim to investigate the involvement of T‐type VGCCs in both endothelium‐dependent and ‐independent relaxant responses in these vessels. Finally, we studied the localization of Ca_V_3.1 and Ca_V_3.2 channels in the aorta and renal artery and their potential co‐localization with endothelial nitric oxide synthase (eNOS) by immunofluorescence.

## 2 METHODS

### 2.1 Ethical approval

All investigators understand the animal ethical principles under which *Experimental Physiology* operates, and this work complies with the journal's animal ethics checklist. All procedures were approved by the Institutional Animal Care and Use Committee of the Universitat de Valencia (Approval Code: 2020/VSC/PEA/0056 type 2) and were conducted in strict compliance with the European Union Directive 2010/63/EU on the protection of animals used for scientific purposes. All steps were taken to minimize the animals' pain and suffering.

### 2.2 Experimental animals and procedures

Fifteen‐week‐old male New Zealand White rabbits (*Oryctolagus cuniculus*) (*n *= 12) with a mean weight of 3.8 ± 0.2 kg were sourced from San Bernardo Laboratories (Navarra, Spain), and housed in individual cages at the Central Research Unit of the Facultat de Medicina i Odontologia, Universitat de Valencia. Housing conditions included a stable temperature of 23°C, and 12‐h light–dark cycles. Rabbits were fed ad libitum. Rabbits were sacrificed by intravenous injection of sodium thiopental (60 mg/kg) with 800 IU of sodium heparin into the marginal vein of the ear. This method is approved for this species and aligns with the standards set out in Annex IV of the European Directive 2010/63/EU. Following euthanasia, abdominal laparotomy was performed to remove the abdominal aorta and renal artery. These artery samples were subsequently used for vascular reactivity and immunofluorescence experiments.

### 2.3 Vascular reactivity study

The aorta and renal artery were mounted immediately after sacrifice in an organ bath for isometric tension recording by introducing two thin, rigid stainless steel wires through the lumen of the vascular segment. One wire was fixed to the wall, while the other was connected to a tension transducer. Isometric tension changes were recorded by a PowerLab data acquisition system (Model PL8/35, ADInstruments, Dunedin, New Zealand) using LabChart 7 software. Each segment was placed in a bath containing 4 mL of Krebs–Henseleit solution (NaCl 115 mM; KCl 4.6 mM; MgCl_2_·6 H_2_O 1.2 mM; CaCl_2_ 2.5 mM; NaHCO_3_ 25 mM; glucose 11.1 mM and EDTA disodium 0.01 mM) equilibrated with a gaseous mixture (95% O_2_ and 5% CO_2_) to maintain a pH of 7.3–7.4. The temperature was kept at 37°C. The optimal resting tension for the arterial segments was 3 g for the aorta and 1 g for renal artery.

Concentration–response curves were constructed for phenylephrine (10^−9^–3 × 10^−5^ M), angiotensin II (10^−11^–3 × 10^−7^ M), acetylcholine (10^−9^–10^−5^ M), and sodium nitroprusside (10^−9^–3 × 10^−6^ M). In the latter two cases, arteries were previously contracted with noradrenaline (10^−7^–10^−6^ M). The involvement of T‐type VGCCs and NO was assessed by incubating arterial rings for 30 min with NiCl_2_ (5 × 10^−5^ M), a T‐type VGCC blocker, and *N* ^ω^‐nitro‐l‐arginine methyl ester (l‐NAME; 10^−4^ M), a nitric oxide synthase (NOS) inhibitor.

In addition, to study whether NO modulates the activity of T‐type VGCCs in the response to phenylephrine, the area under the curve (AUC) was calculated for the concentration–response curves to phenylephrine in the absence and presence of NiCl_2_, l‐NAME and l‐NAME plus NiCl_2_. Then, ΔAUC was determined by subtracting the AUC of the phenylephrine curve incubated with NiCl_2_ from the control phenylephrine curve (AUC 1). This value indicated the involvement of T‐type VGCCs in the absence of l‐NAME. Next, the ΔAUC between phenylephrine curves in the presence of l‐NAME and the curve incubated with l‐NAME plus NiCl_2_ (AUC 2) was also calculated to indicate the involvement of T‐type VGCCs in the presence of l‐NAME. The AUC 1 and AUC 2 values were then compared and plotted as a bar graph to evaluate the participation of T‐type VGCCs in the absence and presence of l‐NAME.

### 2.4 Immunofluorescence in cross‐sections of arteries

Aortic and renal segments (5 mm) were fixed with 4% paraformaldehyde in phosphate‐buffered saline (PBS) (Thermo Fisher Scientific, Waltham, MA, USA) for 1 h at room temperature (RT), followed by three washes with PBS. The fixed arteries were immersed in optimal cutting temperature (OCT) compound and stored at −80°C until cross‐sectioning. Cross‐sections (5 µm thick) were obtained using a cryostat at −20°C, mounted onto poly‐l‐lysine‐coated glass slides, and dried overnight at 4°C. Prior to staining, sections were preincubated for 30 min at RT with a blocking buffer (1% bovine serum albumin, 0.5% Triton X‐100 and 0.05% Tween 20 in PBS). Sections were then incubated with anti‐Ca_V_3.1 (1:100; cat. no. PA5‐99755, Thermo Fisher Scientific) and anti‐Ca_V_3.2 (1:100; cat. no. MA5‐45397, Thermo Fisher Scientific) diluted in blocking buffer overnight at 4°C. After washing, sections were incubated for 30 min at RT with the corresponding secondary antibodies, also diluted in blocking buffer: Alexa Fluor 488 goat anti‐rabbit (1:100; cat. no. A11008, Thermo Fisher Scientific) and Alexa Fluor 647 goat anti‐mouse (1:100; cat. no. A21235, Thermo Fisher Scientific). Nuclei were counterstained with Hoechst 33342 (1:1000; H3570, Thermo Fisher Scientific).

### 2.5 Immunofluorescence of arteries *en face*

Another set of fixed arteries were sliced longitudinally into two pieces for *en face* immunostaining to observe endothelial surface. Tissue pieces were processed in 96‐well culture plates with reagent volumes up to 200 µL/well. Samples were blocked for 2 h *en face* at RT with blocking buffer (1% bovine serum albumin, 0.5% Triton X‐100 and 0.05% Tween 20 in PBS) then incubated with anti‐Ca_V_3.1 (1:100; cat. no. PA5‐99755, Thermo Fisher Scientific), anti‐Ca_V_3.2 (1:100; cat. no. PA5120102, Thermo Fisher Scientific) and anti‐eNOS (1:200; cat. no. ab76198, Abcam, Cambridge, UK) diluted in the same blocking buffer overnight at 4°C. As secondary antibody, Alexa Fluor™ 488 goat anti‐rabbit (1:100; cat. no. A11008, Thermo Fisher Scientific) and Alexa Fluor™ 647 goat anti‐mouse (1:100; cat. no. A21235, Thermo Fisher Scientific) were used at RT for 2 h. Nuclei were counterstained with Hoechst 33342 (1:10,000 dilution, H3570, Thermo Fisher Scientific). For mounting, each piece was carefully positioned on a glass coverslip with the endothelial side facing down and gently flattened using a microscope slide.

### 2.6 Fluorescence imaging

**FIGURE 1:**
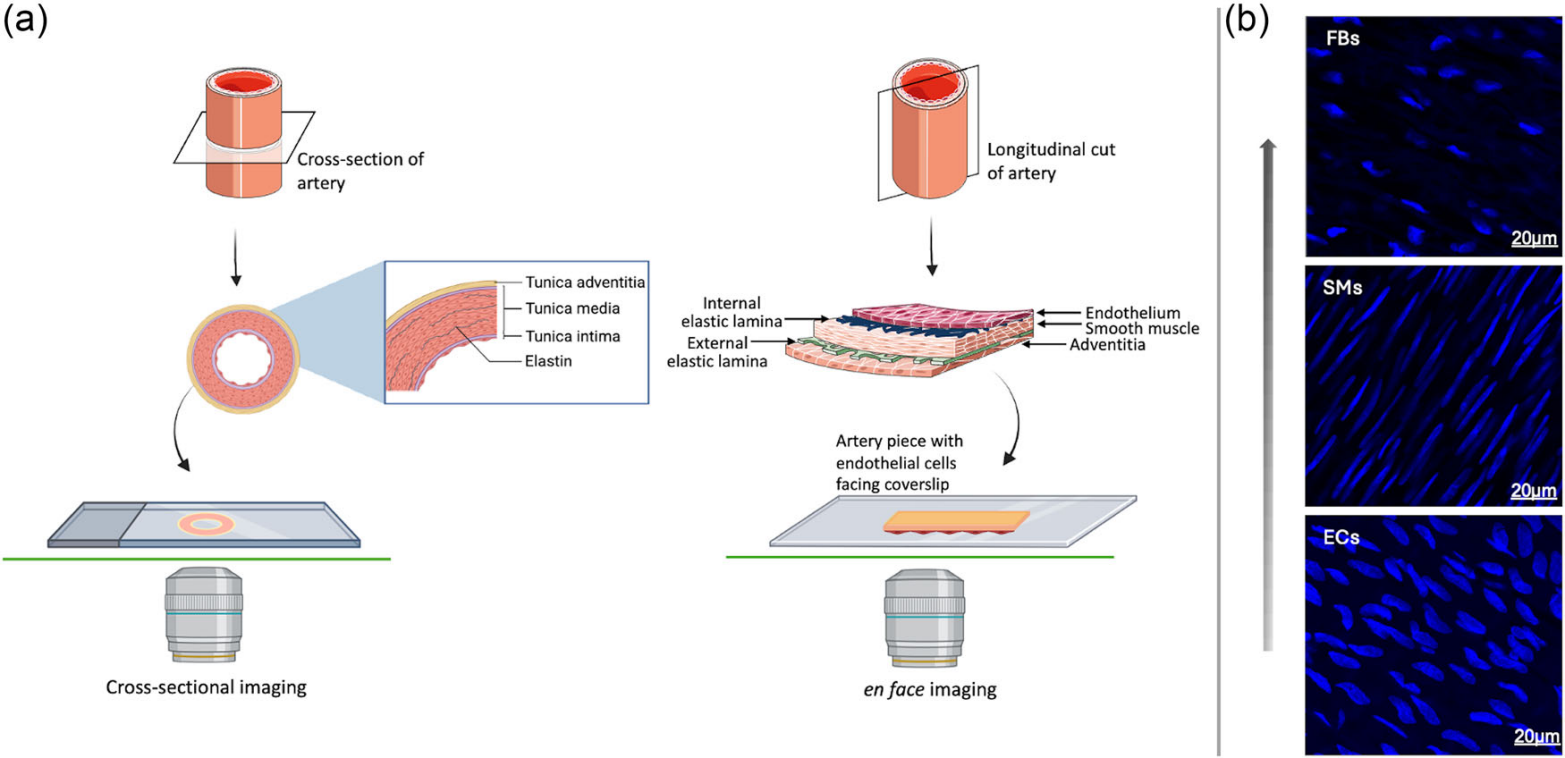
(a) Schematic representation of two preparation techniques for arterial imaging and nuclear morphology of vascular cell types. In the cross‐sectional preparation, the arterial ring is embedded and sectioned transversely to visualize the layered structure of the vessel wall (tunica intima, media and adventitia) on a glass slide. In the *en face* preparation, the artery is opened longitudinally and mounted flat to visualize cells along the luminal surface. (b) Representative confocal images showing nuclear morphology of endothelial cells (ECs), smooth muscle cells (SMs) and fibroblasts (FBs). Nuclei were stained with Hoechst (blue).

Cross‐section and *en face* preparations were mounted in SlowFade Glass Soft‐set (Thermo Fisher Scientific) solution and were imaged using a laser scanning confocal microscope (SP8, Leica, Wetzlar, Germany) with laser lines at 405, 488 and 647 nm for excitation (Figure 1). Z‐stacks were obtained at 1 µm increments (×40 oil‐immersion objective). In the *en face* preparations, scanning along the *z*‐axis enabled sequential visualization of endothelial cells (rounded nuclei and tightly packed), followed by smooth muscle cells (spindle‐shaped nuclei), ending with fibroblasts (elliptical or oval‐shaped nuclei, smaller than endothelial cells), making it possible to distinguish the layers morphologically (Figure 1). Acquired images were analysed using the open‐source software ImageJ/Fiji v2.14.0. The specificity of immunolabelling was tested on preparations without primary antibody.

### 2.7 Colocalization analysis

Colocalization analysis was performed using the Coloc2 plugin in ImageJ/Fiji v2.14.0. to quantitatively assess the spatial relationship between the fluorescence signals of the two markers. Prior to analysis, images were split into individual channels, and regions of interest (ROIs) were selected to avoid background interference. Pearson's correlation coefficient (PCC) was calculated to determine the degree of linear association between the fluorescence intensities of both channels across corresponding pixels, as previously described (Haam et al., 2024). The PCC ranges from −1 to 1, where a value of 1 indicates perfect positive colocalization, −1 reflects complete negative correlation, and 0 denotes the absence of any correlation between the signals. PCC analysis was based on three ROIs per image. A 2D intensity scatter plot was generated to visualize the distribution and correlation of pixel intensities between the two signals, where diagonal clustering indicated areas of strong colocalization.

### 2.8 Drugs and solutions

All drugs for vascular reactivity study were purchased from Sigma‐Aldrich (Merck KGaA, Darmstadt, Germany) and dissolved in purified (MilliQ quality) water. Stock solutions were freshly prepared in saline solution each day and kept on ice during the experiment.

### 2.9 Statistical analysis

Statistical analyses were performed using GraphPad Prism 9.0 (GraphPad Software, Boston, MA, USA). For functional experiments, vasoconstriction was expressed as a percentage of contraction relative to the value of maximal contraction to KCl (60 mM) and vasodilation was expressed as a percentage of relaxation relative to noradrenaline‐induced contraction. The difference of area under the curve (ΔAUC) was calculated for each concentration–response curve to phenylephrine and expressed as arbitrary units. Data normality was assessed using the Shapiro–Wilk test. Multiple comparisons were performed using two‐way analysis of variance (ANOVA) followed by *post hoc* analysis using Bonferroni's test. Differences between two groups were performed using Student's paired two‐tailed *t‐*test. *P *< 0.05 was considered statistically significant. For all experiments *n* represents the number of animals. Data are presented as means ± standard deviation (SD).

## 3 RESULTS

### 3.1 Vascular reactivity

**FIGURE 2:**
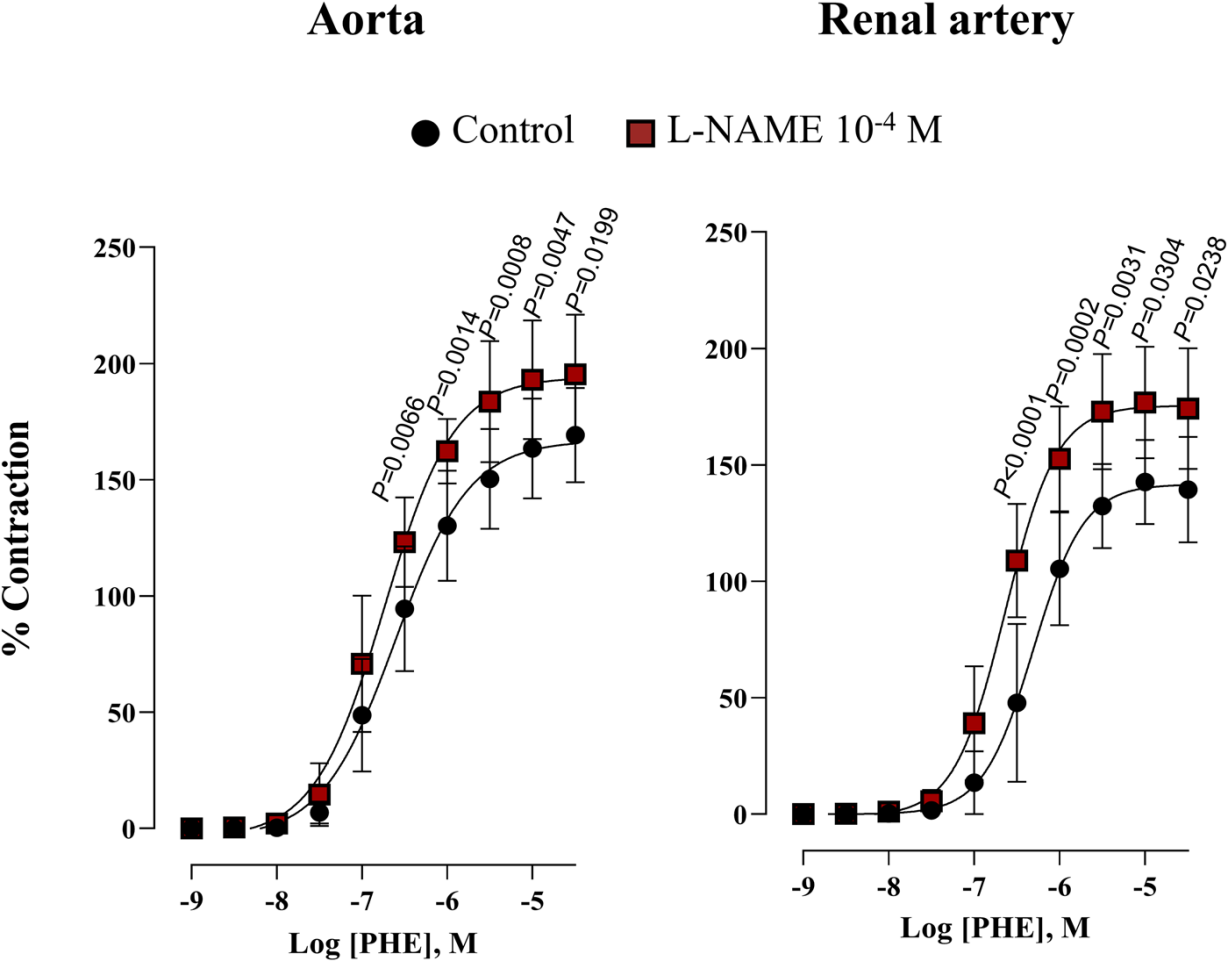
Concentration–response curve to phenylephrine (PHE) in rabbit aorta and renal artery in the absence (control, *n* = 12 for aorta; *n* = 12 for renal artery) and presence of l‐NAME (10^−4^ M, *n* = 9 for aorta; *n* = 12 for renal artery). *n* indicates number of animals. *P*‐values compared to control. Data are means ± SD and were analysed using a two‐way ANOVA followed by a Bonferroni *post hoc* test.

To investigate the modulatory role of NO in vascular α_1_‐adrenergic responsiveness, concentration–response curves to phenylephrine were obtained in the presence and absence of l‐NAME. The significant increase in the contraction in the presence of l‐NAME indicated that endogenous NO exerts a vasodilatory influence on α_1_‐adrenergic‐mediated contraction in both the aorta and renal artery of healthy rabbits (Figure 2).

**FIGURE 3:**
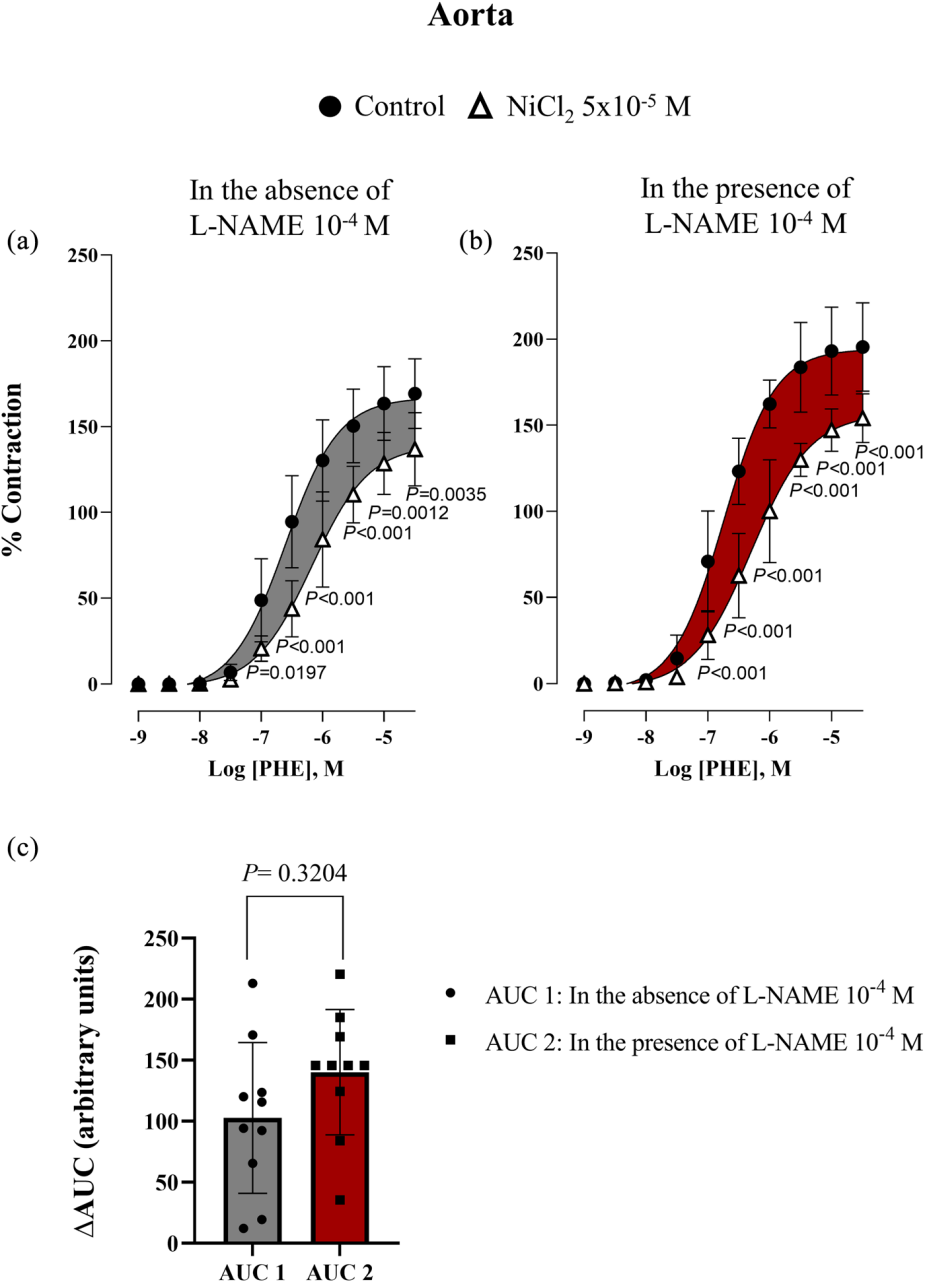
Concentration–response curve to phenylephrine (PHE) in rabbit aorta in the absence (control, n = 12) and presence of NiCl_2_ (5 × 10^−5^ M, n = 7) (a), and in the presence of L‐NAME (10^−4^ M, *n* = 9) and presence of L‐NAME (10^−4^ M) + NiCl_2_ (5 × 10^−5^ M) (*n* = 7) (b). *n* indicates number of animals. *P*‐values compared to control. (c) represents ΔAUC obtained from curves in (a) (grey) and (b) (red), demonstrating the participation of T‐type VGCCs in the response to PHE in the absence and presence of L‐NAME (10^−4^ M). Data are means ± SD and were analysed using a two‐way ANOVA followed by a Bonferroni post hoc test.

**FIGURE 4:**
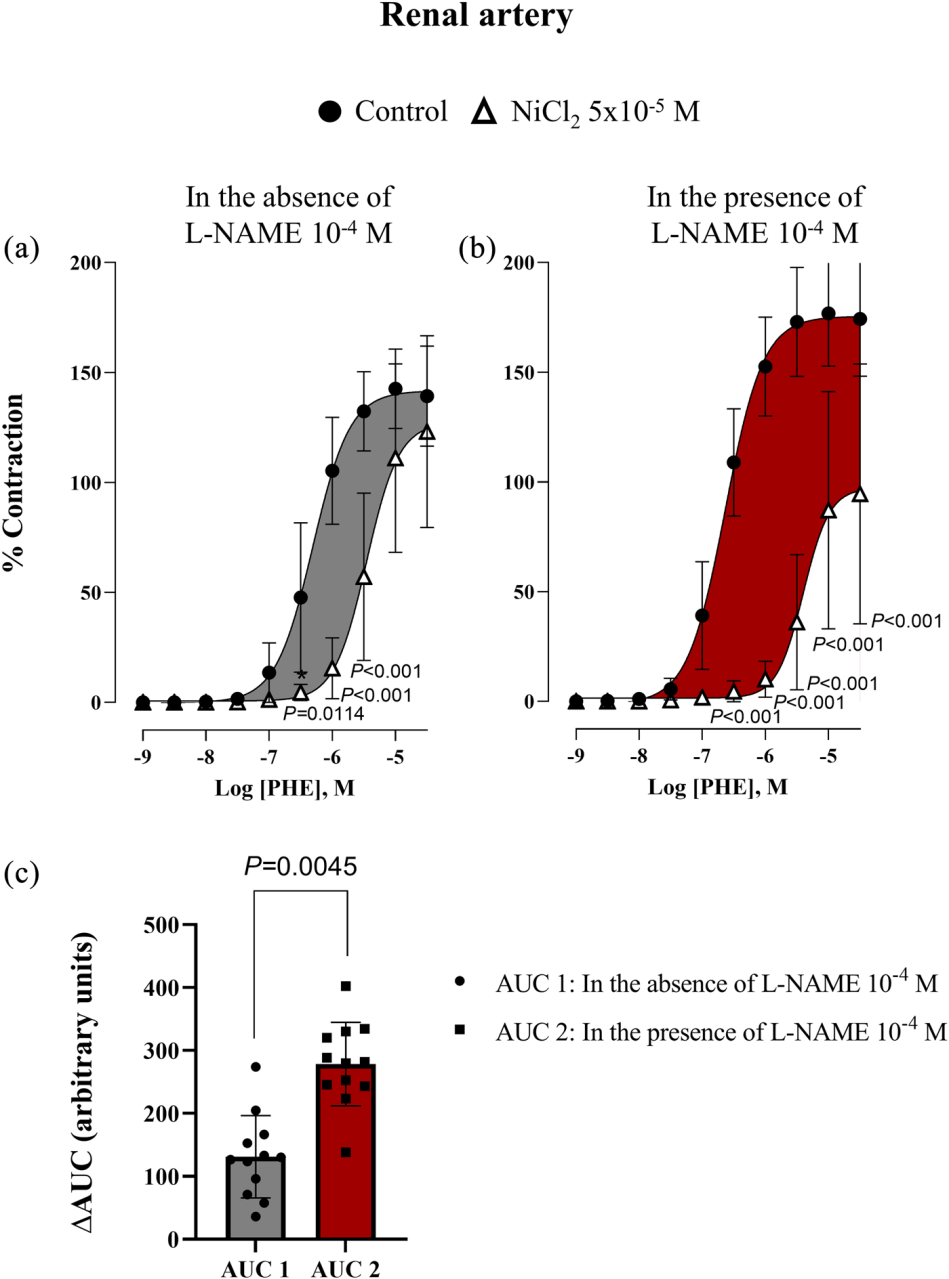
Concentration–response curve to phenylephrine (PHE) in rabbit renal artery in the absence (control, *n* = 12) and presence of NiCl_2_ (5 × 10^−5^ M, *n* = 8) (a), and in the presence of L‐NAME (10^−4^ M, *n* = 12) and presence of L‐NAME (10^−4^ M) + NiCl_2_ (5 × 10^−5^ M) (*n* = 7) (b). *n* indicates number of animals. *P*‐values compared to control. (c) represents ΔAUC obtained from curves in (a) (grey) and (b) (red), demonstrating the participation of T‐type VGCCs in the response to PHE in the absence and presence of L‐NAME (10^−4^ M). Data are means ± SD and were analysed using a two‐way ANOVA followed by a Bonferroni *post hoc* test.

On the other hand, the presence of NiCl_2_ (5 × 10^−5 ^M) induced a rightward shift in the phenylephrine concentration‐curves, indicating the involvement of T‐type VGCCs in α_1_‐adrenergic vasoconstriction in both the aorta (Figure 3a) and renal artery (Figure 4a). To test the hypothesis that NO could modulate the activity of T‐type VGCCs, the concentration–response curves to phenylephrine were performed in the presence and absence of l‐NAME (10^−4 ^M) plus NiCl_2_ (5 × 10^−5 ^M) for aorta (Figure 3b) and renal artery (Figure 4b). Values of pEC_50_ and *E*_max_ of concentration–response curves to phenylephrine in rabbit aorta and renal artery in each experimental condition are presented in Table 1.

**TABLE 1:** Values of pEC_50_ and *E*_max_ of concentration–response curves to phenylephrine in rabbit aorta and renal artery in the absence (control) and presence of l‐NAME (10^−4^ M), nickel chloride (NiCl_2_) (5 × 10^−5^ M), or the combination of both.

	*n*	pEC_50_	*P*	*E*_max_	*P*
Aorta					
Control	12	6.55 ± 0.27		162.25 ± 30.83	
l‐NAME 10^−4^ M	9	6.82 ± 0.27	0.0330*	196.56 ± 9.13	0.0160
NiCl_2_ 5 × 10^−5^ M	7	6.20 ± 0.20	0.0092*	135.14 ± 19.05	0.0518*
l‐NAME 10^−4^ M + NiCl_2_ 5 × 10^−5^ M	7	6.29 ± 0.36	0.0041^†^	154.00 ± 14.21	0.0023^†^
Renal artery					
Control	12	6.34 ± 0.26		145.08 ± 19.03	
l‐NAME 10^−4^ M	12	6.64 ± 0.20	0.0053*	176.08 ± 26.31	0.0032*
NiCl_2_ 5 × 10^−5^ M	8	5.56 ± 0.40	0.00005*	125.13 ± 42.54	0.1677
l‐NAME 10^−4^ M + NiCl_2_ 5 × 10^−5^ M	7	5.40 ± 0.16	<0.00001^†^	96.71 ± 61.52	0.00103^†^

Data are means ± SD. *n* = number of animals. *P‐values compared to control; ^†^ *P*‐values compared to l‐NAME 10^−4^ M.

The results showed that in the presence of l‐NAME, the contribution of T‐type VGCCs in the contractile response to phenylephrine was not altered in the aorta since we did not find differences between AUC 1 and AUC 2 (149.20 ± 27.02 vs. 110.20 ± 28.22, for AUC 2 and AUC 1 respectively; *P *= 0.3204, Figure 3c). However, in the renal artery, AUC 2 was significantly increased compared to AUC 1 (278.3 ± 33.8 vs. 131.00 ± 30.56, for AUC 2 and AUC 1 respectively; *P *= 0.0045) indicating that in the presence of l‐NAME, the contribution of T‐type VGCCs was enhanced (Figure 4c).

**FIGURE 5:**
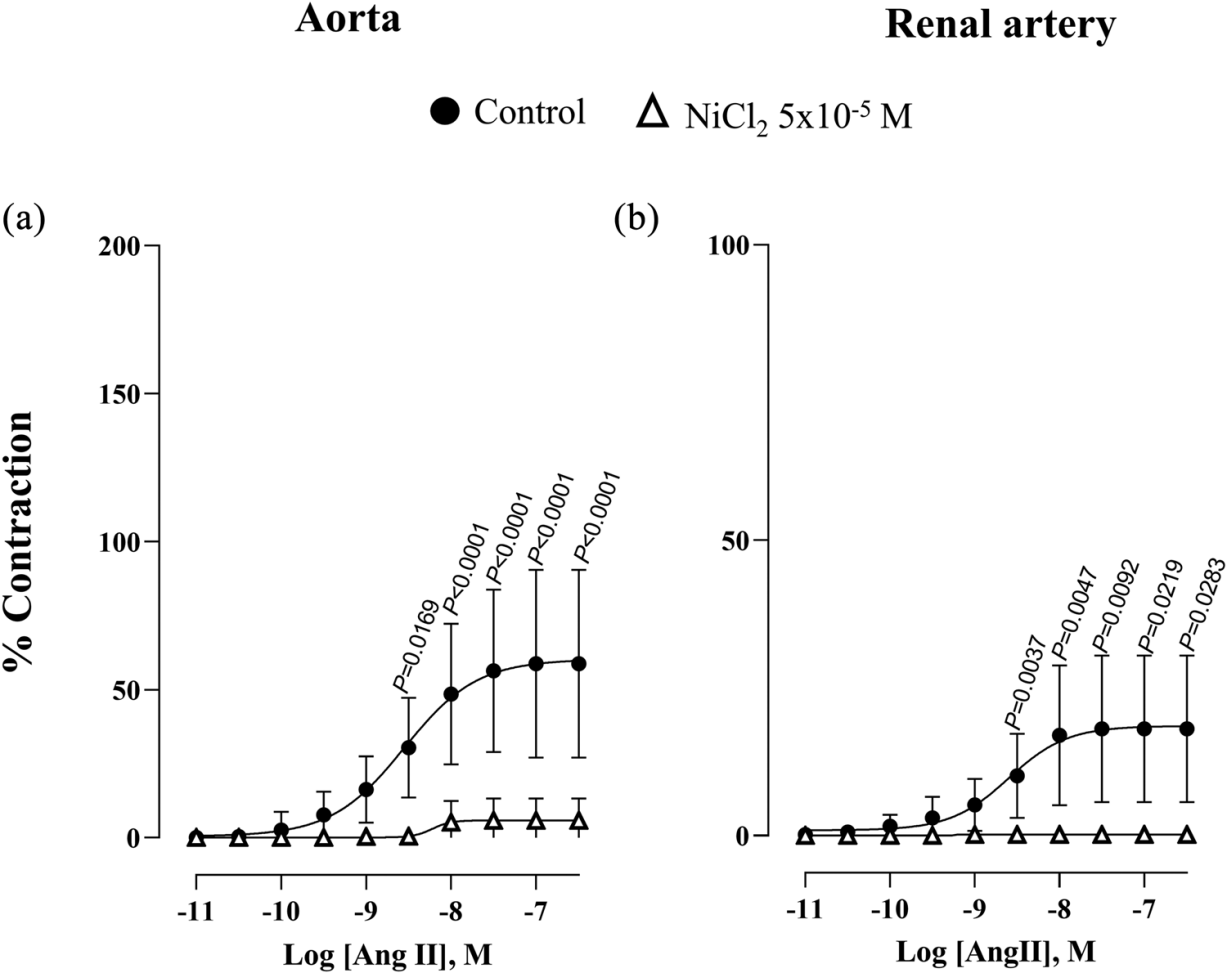
Concentration–response curve to angiotensin II (Ang II) in rabbit aorta and renal artery in the absence (control *n* = 11 for aorta; *n* = 10 for renal artery) and presence of NiCl_2_ (5 × 10^−5^ M, *n* = 6 for aorta; *n* = 6 for renal artery). *n* indicates number of animals. *P*‐values compared to curve in the presence of NiCl_2_. Data are means ± SD and were analysed using a two‐way ANOVA followed by a Bonferroni *post hoc* test.

The presence of NiCl_2_ (5 × 10^−5 ^M) significantly right‐shifted the concentration–response curve to angiotensin II, abolishing the contraction. This indicates that Ca^2+^ influx through T‐type VGCCs plays a key role in angiotensin II‐induced contraction in the aorta and renal artery of healthy rabbits (Figure 5).

**FIGURE 6:**
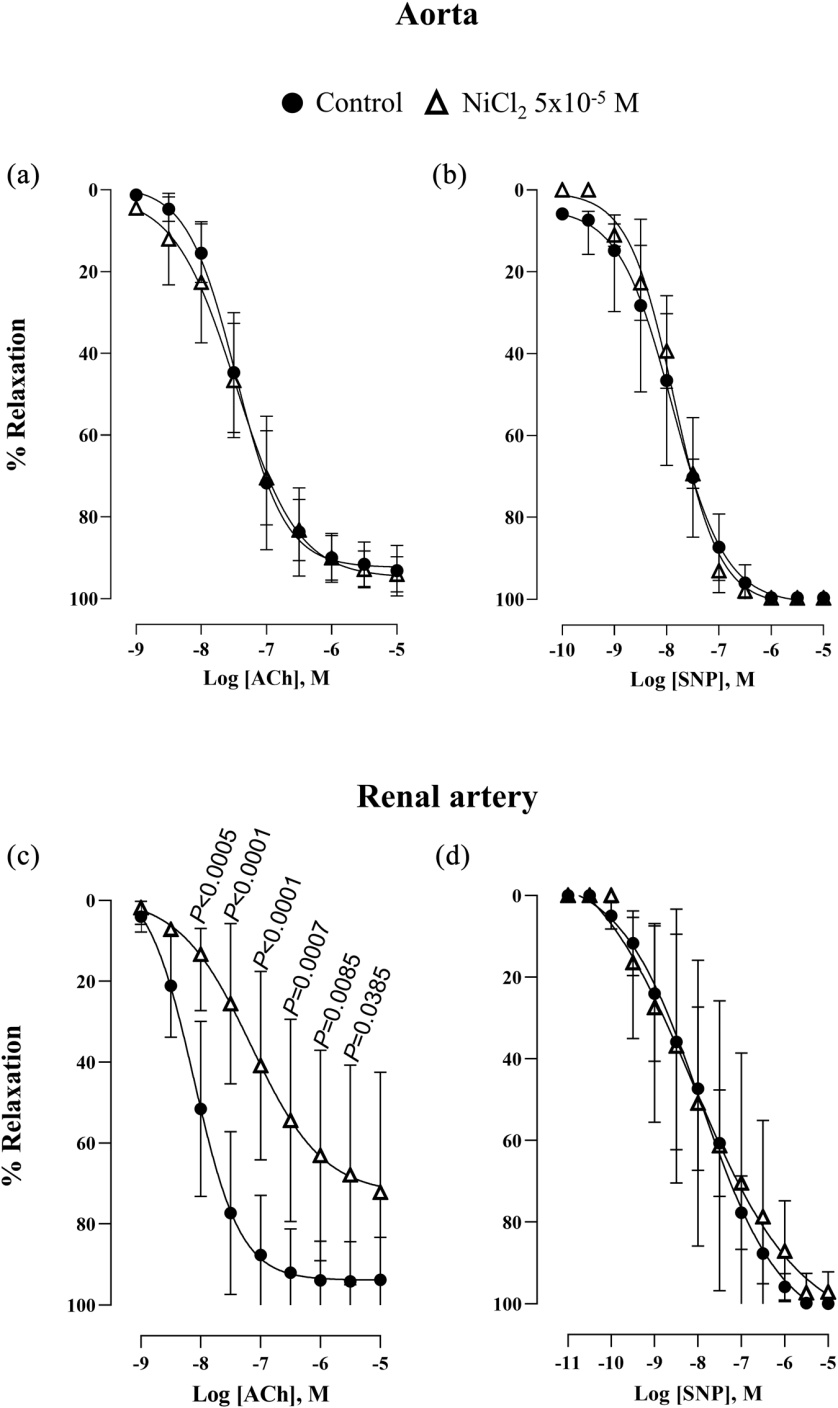
Concentration–response curve to acetylcholine (ACh) (a, c) and to sodium nitroprusside (SNP) (b, d) in rabbit aorta and renal artery in the absence (control, *n* = 9 for aorta ACh, *n* = 9 for aorta SNP, *n* = 9 for renal ACh and *n* = 9 for renal SNP) and presence of NiCl_2_ (5 × 10^−5^ M, n = 6 for aorta ACh, *n* = 5 for aorta SNP, *n* = 6 for renal ACh and *n* = 7 for renal SNP). *n* indicates number of animals. *P*‐values compared to control. Data are means ± SD and were analysed using a two‐way ANOVA followed by a Bonferroni *post hoc* test.

In addition, we investigated the role of T‐type VGCCs in both endothelium‐dependent and ‐independent vasodilation. In the aorta, NiCl_2_ (5 × 10^−5 ^M) did not affect the relaxation induced by acetylcholine or sodium nitroprusside (Figure 6a, b). However, in the renal artery, NiCl_2_ reduced the response to acetylcholine but had no effect on the response to sodium nitroprusside (Figure 6c, d).

**FIGURE 7:**
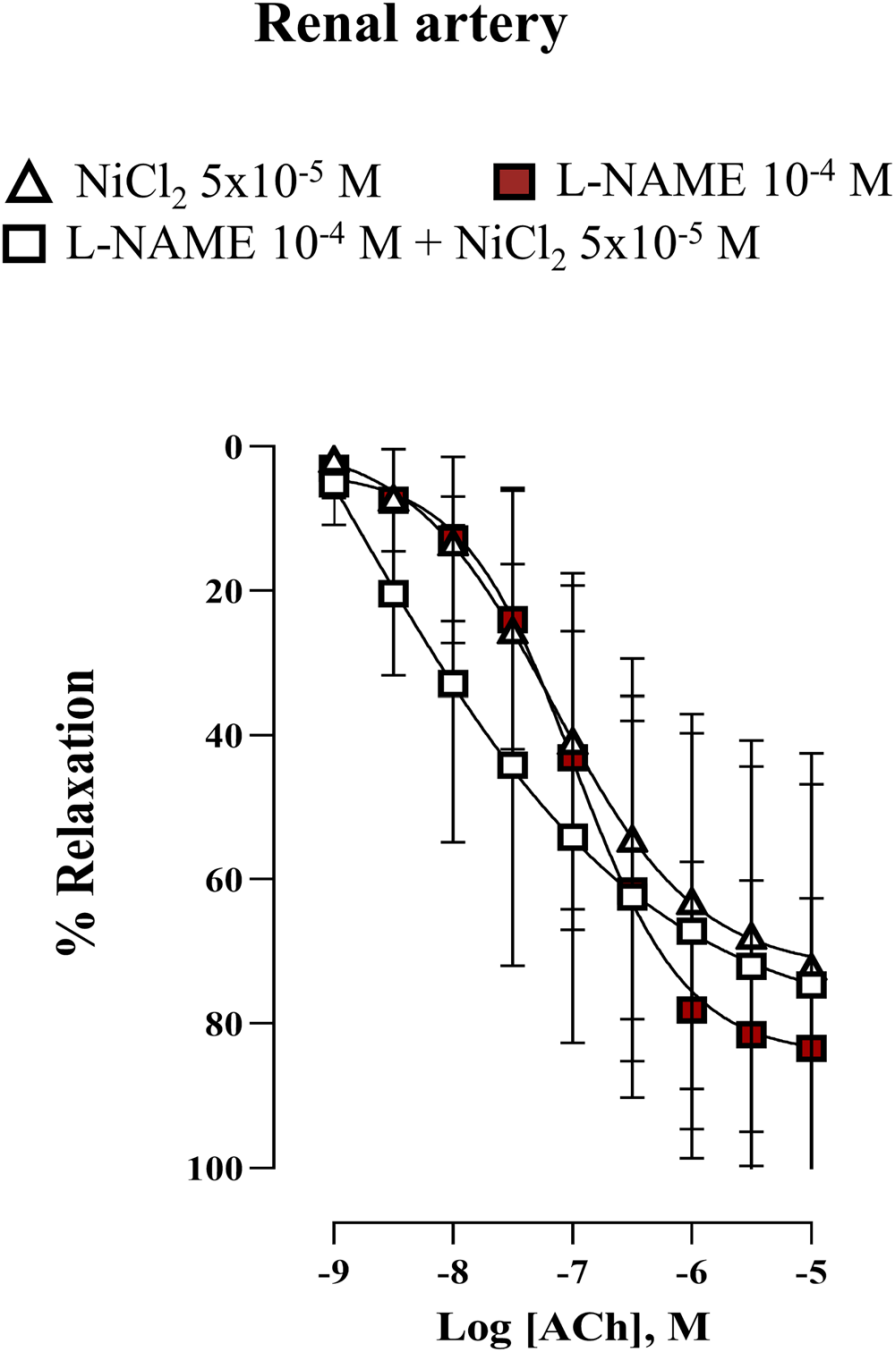
Concentration–response curve to acetylcholine (ACh) in rabbit renal artery in the presence of l‐NAME (10^−4 ^M, *n* = 9), NiCl_2_ (5 × 10^−5^ M, *n* = 6) and co‐incubation of l‐NAME (10^−4 ^M) plus NiCl_2_ (5 × 10^−5^ M) (*n* = 8). *n* indicates number of animals. Data are means ± SD and were analysed using a two‐way ANOVA followed by a Bonferroni *post hoc* test.

The blockade caused by NiCl_2_ on acetylcholine response was similar to that induced by l‐NAME, and co‐incubation of l‐NAME plus NiCl_2_ did not show a synergic blockade compared to that induced by NiCl_2_ or l‐NAME alone (Figure 7).

**FIGURE 8:**
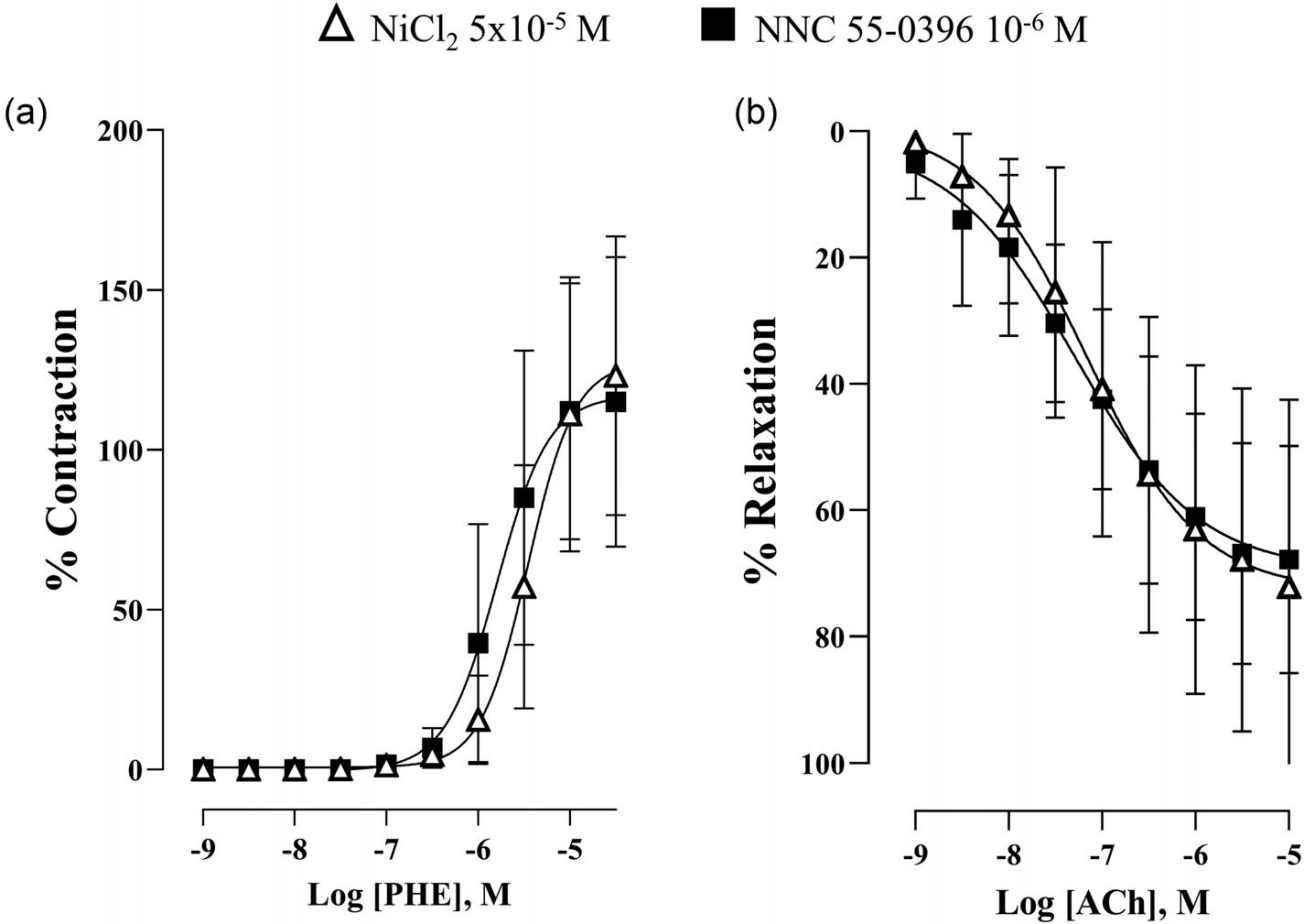
Concentration–response curve to phenylephrine (Phe) (a) and acetylcholine (ACh) (b) in the rabbit renal artery in the presence of NiCl_2_ (5 × 10^−5^ M, *n* = 7 for Phe, *n* = 6 for ACh) and NNC 55‐0396 (10^−6^ M, *n* = 5 for Phe, *n* = 5 for ACh). *n* indicates number of animals. Data are shown as means ± SD and were analysed using a two‐way ANOVA followed by a Bonferroni *post hoc* test.

NiCl_2_ is a T‐type VGCC blocker which may exert off‐target effects on other ion channels and exchangers. To address this limitation, we compared the effects of NiCl_2_ with those of NNC 55‐0396, a more selective T‐type VGCC blocker. Both the vasocontractile and vasodilatory responses observed with NiCl_2_ (5 × 10^−^⁵ M) were similar to those obtained with NNC 55‐0396 (10^−^⁶ M) (Figure 8). These comparable results support the interpretation that the effects observed with NiCl_2_ are indeed attributable to T‐type VGCC blockade.

### 3.2 Expression of Ca_V_3.1 and Ca_V_3.2 in aorta and renal artery

**FIGURE 9:**
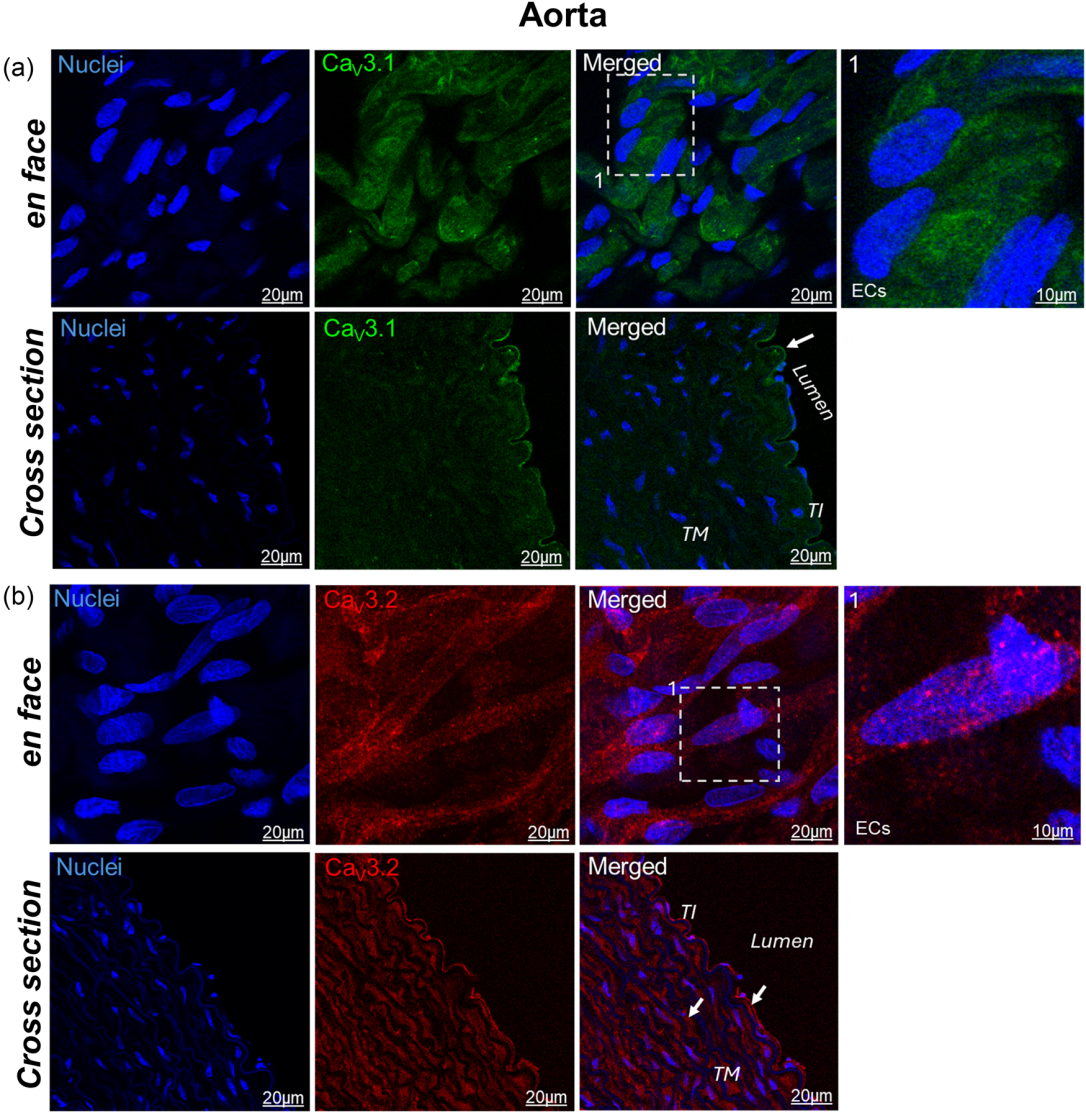
Representative confocal laser‐scanning microscopy images of rabbit aorta stained for Ca_V_3.1 (green) (a) and Ca_V_3.2 (red) (b). Nuclei were stained with Hoechst (blue). White dashed box in the merge images is amplified at the right column. White arrows indicate positive expression. Images are representative of 3 independent experiments. ECs, endothelial cells; TI, tunica intima; TM, tunica media.

Confocal laser‐scanning microscopy revealed robust Ca_V_3.1 expression in the endothelium of the aorta, with no detectable signal in the smooth muscle layer (Figure 9a). In contrast, Ca_V_3.2 expression was detected in both the endothelium and smooth muscle (Figure 9b).

**FIGURE 10:**
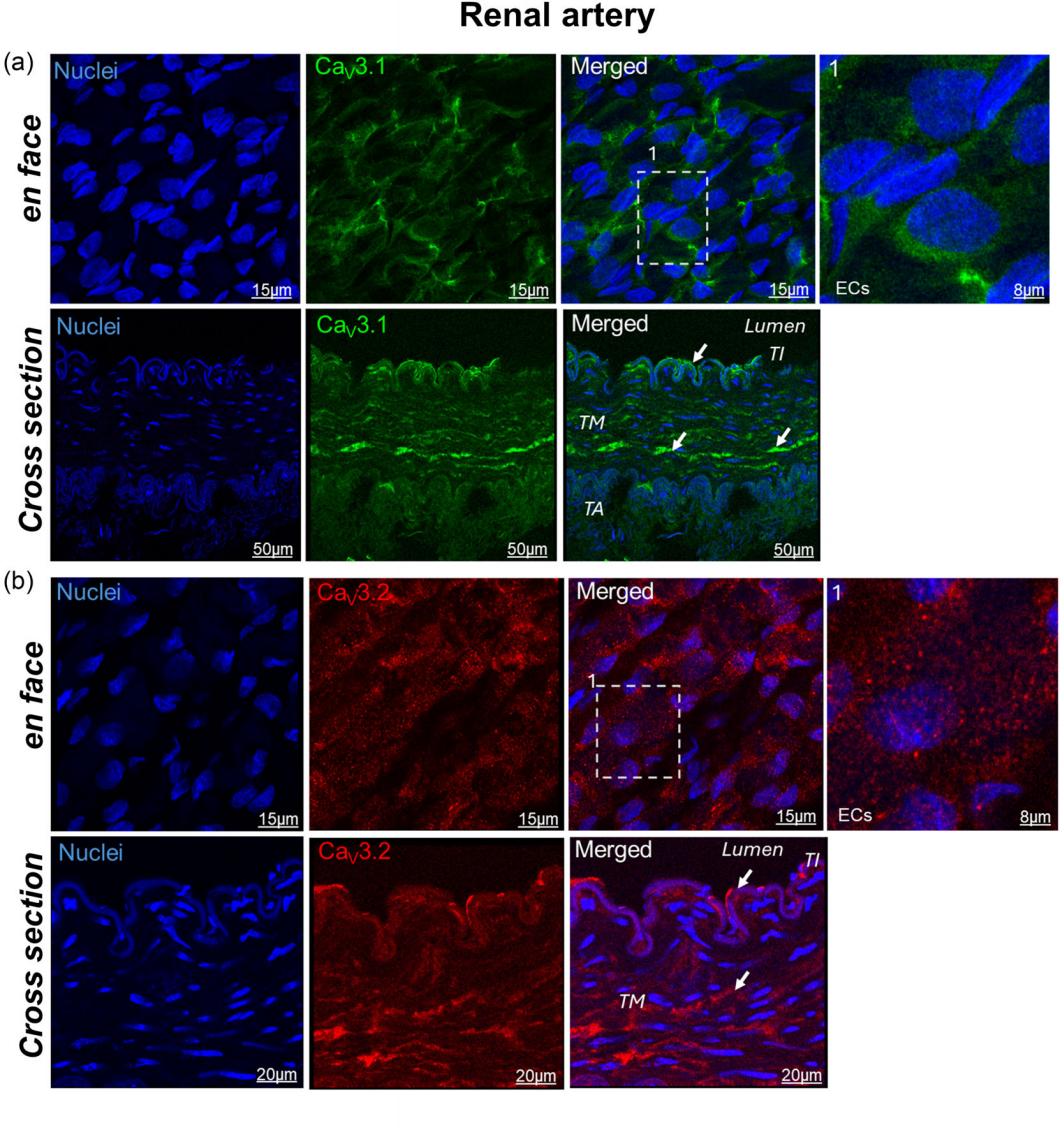
Representative confocal laser‐scanning microscopy images of rabbit renal artery stained for Ca_V_3.1 (green) (a) and Ca_V_3.2 (red) (b). Nuclei were stained with Hoechst (blue). White dashed box in the merge images is amplified at the right column. White arrows indicate expression. Images are representative of three independent experiments. ECs, endothelial cells; TA, tunica adventitia; TI, tunica intima; TM, tunica media.

In the renal artery, both Ca_V_3.1 and Ca_V_3.2 were expressed in the endothelium as well as in the smooth muscle (Figure 10). Ca_V_3.2 exhibited a characteristic punctate staining pattern in both vascular beds.

**FIGURE 11:**
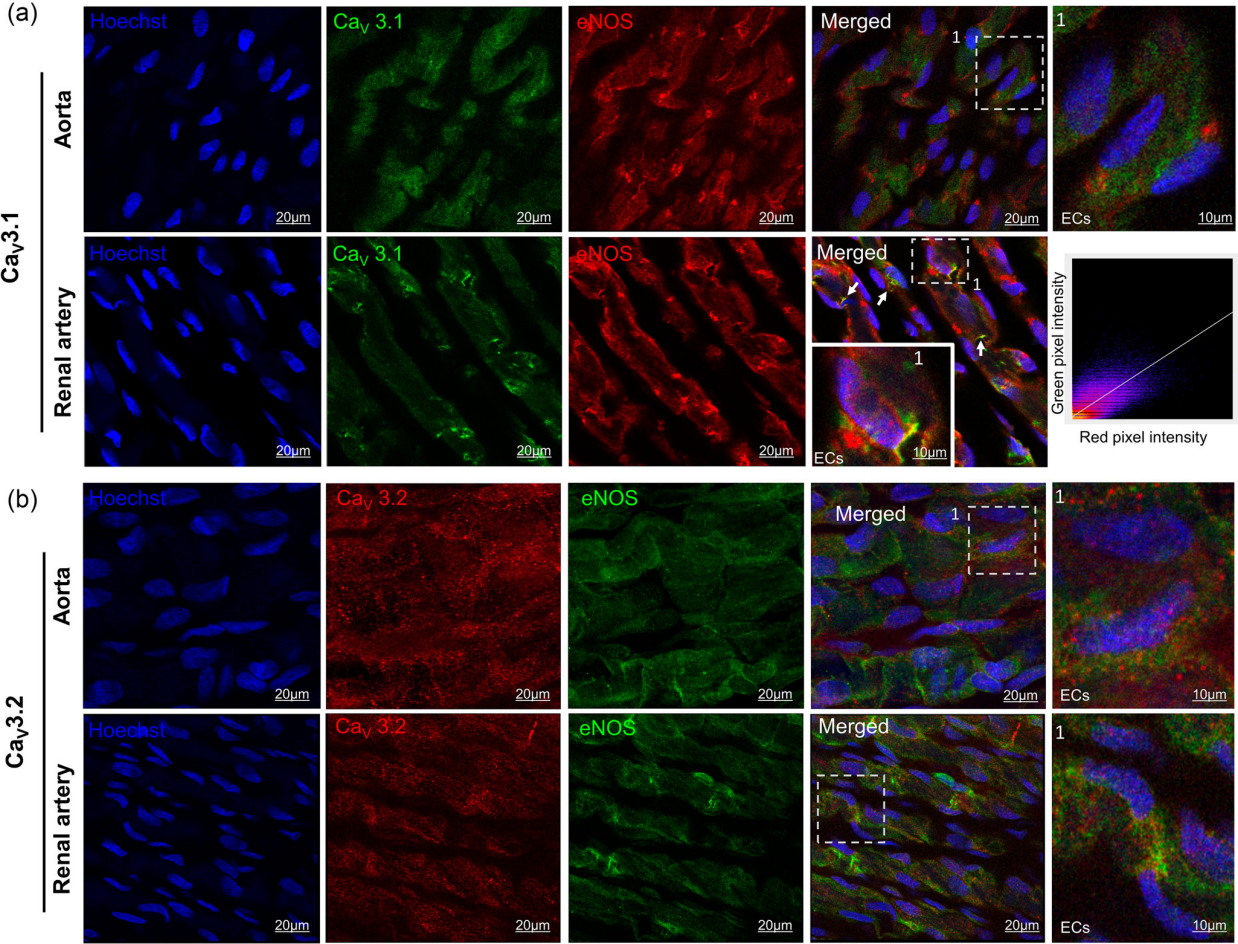
Representative confocal laser‐scanning microscopy image of rabbit aorta and renal endothelial cells stained for Ca_V_3.1(green)/eNOS (red) (a) and Ca_V_3.2 (red)/eNOS (green) (b). Nuclei were stained with Hoechst (blue). The white dashed box in the merged images is shown magnified in the right‐hand panels. White arrows indicate areas of co‐localization (yellow signal). Co‐localization was quantified within the selected ROI (white dashed box in the magnified Ca_V_3.1 image from the renal artery) using the Coloc2 plugin in ImageJ/Fiji and visualized through a scatter plot displaying green and red channel intensities on the *y*‐ and *x*‐axes, respectively. Images are representative of three independent experiments. ECs, endothelial cells.

To investigate potential functional associations, we examined the colocalization between T‐type VGCC subtypes and eNOS. Interestingly, a clear spatial overlap between endothelial Ca_V_3.1 and eNOS was observed exclusively in the renal artery, whereas no such colocalization was detected in the aorta (Figure 11). This observation was further supported by a quantitative colocalization analysis, which included a 2D scatter plot (Figure 11) showing a significant positive linear correlation between the fluorescence intensities of Ca_V_3.1 and eNOS. The calculated Pearson's correlation coefficient was 0.71 ± 0.024, indicating a moderate to high degree of colocalization, and suggesting that the structures labelled in both channels exhibit a similar spatial distribution within the analysed ROIs. These findings may support a physiological role for Ca_V_3.1 in eNOS activation within the renal artery, potentially by mediating localized calcium influx necessary for enzyme activation.

## 4 DISCUSSION

The regulation of vascular tone by T‐type VGCCs is complex. Initial studies suggested a functional dichotomy between the Ca_V_3.1 and Ca_V_3.2 subtypes, with the former linked to vasoconstriction and the latter to vasodilation (Hansen, 2015). However, knockout models have shown that both subtypes may participate in both vasoconstrictive and vasodilatory mechanisms (Hansen, 2015). In the present study, we report that T‐type VGCCs are expressed in large conduit arteries, including the aorta and renal artery, and that their functional contribution to vascular tone is artery‐specific.

NiCl_2_ has been reported to be relatively selective for Ca_V_3.2 at a concentration of 50 µM (Kang et al., 2006) over other T‐type channels. However, the study by Obejero‐Paz et al. (2008) provides clear evidence that NiCl_2_ at 10–100 µM is capable of blocking Ca_V_3.1 channels. Therefore, the findings observed in our study could be due to the blockade of both Ca_V_3.1 and Ca_V_3.2 subtypes. Moreover, NiCl_2_ may exert off‐target effects on other ion channels and exchangers (Melgari et al., 2022). To address this limitation, we compared the effects of NiCl_2_ with those of NNC 55‐0396, a more selective T‐type VGCC blocker (Huang et al., 2004), and the comparable results support the interpretation that the effects observed with NiCl_2_ are indeed attributable to T‐type VGCC blockade.

Our findings demonstrate that T‐type VGCCs contribute to α_1_‐adrenergic and angiotensin II‐induced vasoconstriction in the rabbit aorta and renal artery under physiological conditions. Notably, the blockade of T‐type VGCCs completely abolished angiotensin II response. This suggests that angiotensin II‐induced contraction relies predominantly on calcium influx via T‐type VGCCs in the aorta and renal artery. Given that blockade of angiotensin II effects is a cornerstone of antihypertensive therapy, this finding underscores a potential mechanistic link between T‐type VGCC activity and the regulation of blood pressure. However, further experiments are necessary to clarify the relationship between T‐type VGCCs and angiotensin II, as other authors (Thuesen et al., 2019) observed that Ca_V_3.1 and Ca_V_3.2 knockout mice, in which hypertension was induced by angiotensin II infusion, behaved similarly to wild‐type mice; that is, the increase in mean arterial pressure was the same across all three groups. It was observed that angiotensin II‐induced aldosterone secretion was attenuated in Ca_V_3.1 knockout mice, confirming distinct roles for Ca_V_3.1 and Ca_V_3.2 channels in response to angiotensin II. Our study in rabbits suggests a direct vascular role for T‐type VGCCs in response to angiotensin II, whereas the study performed by Thuesen et al. (2019) in knockout mice points to a differential endocrine role, particularly in aldosterone secretion.

To investigate the interaction between T‐type VGCCs and NO signalling, we evaluated the α_1_‐adrenergic response in the presence of l‐NAME, an eNOS inhibitor. In both arteries, phenylephrine‐induced NO release attenuated contractile responses. Dual blockade with NiCl_2_ and l‐NAME revealed an enhanced contribution of T‐type VGCCs in the renal artery under NO‐deficient conditions, whereas no such effect was observed in the aorta. These findings suggest a vessel size‐dependent role for T‐type VGCCs, with their contribution increasing as vessel diameter decreases (Abd El‐Rahman et al., 2013; Ball et al., 2009; Jensen & Holstein‐Rathlou, 2009; Kuo et al., 2010). Moreover, eNOS inhibition may augment T‐type VGCC function, as previously reported in cerebral and mesenteric arteries (Howitt et al., 2013, 2015; Ng et al., 2024; Smith et al., 2020). Hence, reduced NO bioavailability, associated with endothelial dysfunction and cardiovascular diseases (Roy et al., 2023), may enhance the contribution of T‐type VGCCs, particularly in renal circulation, as suggested by our results, where the role of these channels appears to be relevant (Feng et al., 2004). Extending these observations to a clinical setting, recent evidence in healthy 60‐year‐old men demonstrated that 8 weeks of treatment with efonidipine, a dual L‐ and T‐type VGCC blocker, improved endothelium‐dependent vasodilation. This effect was not observed with nifedipine, which selectively blocks L‐type VGCCs, suggesting that T‐type VGCCs might be key in age‐related endothelial dysfunction (Iepsen et al., 2024). However, based on the literature available to date, it is highly likely that the blockade of T‐type VGCCs did not have a direct effect on mean arterial pressure (Chiang et al., 2009; Hansen, 2015; Harraz et al., 2015; Iepsen et al., 2024; Thuesen et al., 2014, 2019). As demonstrated in our study the role of T‐type VGCCs would rather consist in modulating the mechanisms at the vascular level. Indeed, these channels may also have a vasodilatory role, as observed in the renal artery of healthy rabbits, where the Ca_V_3.1 subtype is specifically required for endothelium‐dependent vasodilation. Ca_V_3.1 triggers an increase in intracellular Ca^2+^ concentration in the vicinity of eNOS, which could activate the enzyme and promote vasodilation (Svenningsen et al., 2014). To test this hypothesis, we performed experiments in the presence of l‐NAME, where the inhibition of acetylcholine‐induced vasodilation was comparable to that observed with NiCl_2_. Furthermore, co‐incubation with both l‐NAME and NiCl_2_ did not produce additive inhibitory effects, suggesting that T‐type VGCC‐mediated vasodilation operates through the same NO‐dependent pathway. These findings support the notion that the calcium influx required to activate eNOS in the rabbit renal artery occurs via T‐type VGCCs, particularly endothelial Ca_V_3.1. This is further supported by our evidence of Ca_V_3.1 and eNOS spatial colocalization, which was not detected in the aorta. In the pulmonary circulation, it has been proposed that acetylcholine can activate T‐type VGCCs (Gilbert et al., 2017). One of the potential mechanisms involves modulation of the resting membrane potential. Given that the resting membrane potential of endothelial cells is relatively high (approximately −45 mV), T‐type VGCCs are typically inactivated. Acetylcholine may hyperpolarize endothelial cells by activating potassium channels, and as a result, this hyperpolarization could shift the membrane potential into the activation range of T‐type VGCCs, thereby allowing Ca^2^⁺ influx, NO release, and vasodilation.

The insightful study by Thuesen et al. (2014) using Ca_V_3.1 and Ca_V_3.2 knockout mice suggests a vasoconstrictive role for Ca_V_3.1 in renal afferent arterioles and a vasodilatory role for Ca_V_3.2 in renal efferent arterioles. In their model, Ca_V_3.1 knockout mice exhibited increased renal plasma flow, while Ca_V_3.2 knockout mice showed elevated glomerular filtration rate. However, our study in rabbit renal artery suggests that Ca_V_3.1 may exert a vasodilatory effect, based on its colocalization with eNOS and the reduced response to acetylcholine following T‐type VGCC blockade. These apparent discrepancies may be attributed to differences in experimental design. Notably, Thuesen et al. (2014) investigated the effects of T‐type VGCCs in the renal microcirculation, whereas our study focused on the main renal artery. Thuesen et al. assessed systemic and renal effects of T‐type VGCCs in an in vivo model, while our study evaluated *ex vivo* vascular function in healthy rabbit renal arteries. *Ex vivo* studies eliminate neural and hormonal influences present in in vivo models, thereby revealing local vascular responses. Supporting this notion, Thuesen et al. reported no significant differences in the contractility of isolated arterioles and suggested that the observed effects may be mediated by neurogenic or systemic mechanisms. This finding further highlights the complexity of the role played by these channels.

Since our study focuses on isolated blood vessels, extrapolating our findings to humans requires further investigation. In this paper, we demonstrate that T‐type VGCCs do not modulate vasodilation in the aorta but facilitate it in the renal artery. In contrast, other authors have reported that in the human femoral artery, combined blockade of L‐type and T‐type VGCCs enhances endothelium‐dependent vasodilation (Iepsen et al., 2024). These discrepancies may be attributed to differences in vascular beds as well as methodological approaches, given that our study utilizes *ex vivo* vessels, whereas Iepen's work involves acetylcholine infusion into the femoral artery in vivo. Additional studies are necessary to deepen our understanding of the functional role of these channels and to identify potential clinical applications.

The present study is not without limitations. Our findings on Cav3.1 and eNOS colocalization in the renal artery provide only indirect evidence of spatial association, warranting cautious interpretation until direct molecular interactions and mechanisms are confirmed. In addition, the exclusive use of male rabbits limits generalizability by precluding the assessment of sex‐specific differences in vascular responses. Future studies addressing both constraints, including the incorporation of both sexes, will be critical to fully elucidate the role of T‐type VGCCs in vascular physiology.

In summary, our data reveal that T‐type VGCCs are functionally expressed in large conduit arteries, including the aorta and renal artery, extending their known role beyond small resistance vessels. Angiotensin II‐mediated contraction is primarily facilitated through Ca2^+^ influx via T‐type VGCCs, which also play a role in the contractile response to phenylephrine. In the absence of NO, T‐type VGCCs may become more active only in the renal artery, where they exhibit both contractile and relaxing functions, Ca_V_3.1 being necessary in the NO‐mediated vasodilation. In the aorta, these channels have a predominantly contractile function. Therefore, renovascular T‐type VGCCs might provide novel therapeutic targets, but the use of T‐type blockers should be specifically directed to the channel subtype. The differential expression and functional roles of these channels across vascular territories highlight the complexity of calcium signalling in vascular physiology. Our findings provide new insights into the function and modulation of T‐type VGCCs under physiological conditions, and their relationship with NO, shedding light on their critical role in regulating vascular tone.

## AUTHOR CONTRIBUTIONS

Maria D. Mauricio and Eva Serna designed the study; Andrea Suarez, Sol Guerra‐Ojeda, Manuel Zarzoso, Marta Serna‐García., and Jose M. Vila acquired the data; Andrea Suarez, Sol Guerra‐Ojeda, Eva Serna and Maria D. Mauricio analysed the data; Andrea Suarez and Maria D. Mauricio wrote the initial draft of the manuscript. All authors revise it critically for important intellectual content. All authors read and approved the final version of the manuscript and agree to be accountable for all aspects of the work in ensuring that questions related to the accuracy or integrity of any part of the work are appropriately investigated and resolved. All persons designated as authors qualify for authorship, and all those who qualify for authorship are listed.

## CONFLICT OF INTEREST

None declared.

## Data Availability

All data supporting the findings of this study are available within the manuscript itself.
